# Personal

**Published:** 1912-07

**Authors:** 


### PERSONAL

Dr. Mott Moore and Dr. Joseph Roby are in charge of the Rochester Infants' Summer Hospital, situated on the lake shore at Charlotte. The staff recently appointed consists of Drs. Orchard, Witherspoon, Wesley, Mulligan, E. W. Mulligan, McDowell, Hanes, Fitch, Ruggles, Snell, and Richards.

---

Dr. Edward B. Angell made the address to the graduating class of the nurses' training school of the Rochester State Hospital, June 12, 1912. The class consisted of 11 women and 1 man. Dr. E. H. Howard, Superintendent, and Col W. C. Sanger and Dr. J. V. May, of the State Hospitals Commission spoke to the class and presented the diplomas.

---

Dr. Spencer L. Dawes, of the special commission named by the Governor to investigate the alien insane situation, took testimony at the Willard State Hospital on June 11 and at the Rochester State Hospital on June 12, 1912.

---

Dr. Leonard W. Jones has removed his office to 53 S. Fitzhugh St., Rochester.

---

The property of the Denniston (automobile) Co. has recently been sold to Dr. E. J. Meyer of Buffalo.

---

On June 27, 1912, Dr. Joseph S. Lewis of Buffalo, was amrried to Miss Isabelle St. Clair Denny, at Charlotte, N. Y.

---

The recent collapse of a dock at Eagle Park, Grand Island, near Buffalo, resulted in about 40 deaths by drowning. Dr. George B. Stocker, the post mortem examiner, rendered efficient service. Dr. Frederick E. Sperry of Florida St., Buffalo and Dr. M. C. Vaughan an interne in the Buffalo Hospital of the Sisters of Charity, helped to save lives. Charles A, Seward, an interne at the State Hospital, Buffalo, nearly lost his own life insaving his wife and child.

---

Mr. August Schneider, clerk of the Buffalo Health Department, has retired from the position of Drum Major of the 74th Regiment, receiving an honorary lieutenancy. He is succeeded in the regiment by William Witte formerly of the Kilties' Band. Mr. Witte is seven feet, two inches in height, and is one of the tallest men in the world, if not the tallest drum-major. Just the same, a new generation will have to grow up before Buffalo will cease to miss Mr. Schneider. Our best wishes to him and apologies for designating him other than more familiar and endearing nick-name.

---

Dr. Max Carl Breuer of Buffalo, and family, will spend the summer in European travel.

---

Dr. John R. Williams of Rochester, whose departure for Europe was announced in the June issue, informs us that pressure of work has made it necessary to postpone his trip till next year.

---

Dr. S. P. Jewett has recently opened an office on West Chippewa St., near Delaware Ave., Buffalo.

---

Dr. A. E. Forsyth of Buffalo, was in New York late in June.

---

Dr. Otto R. Eichel of Buffalo, has been appointed Superintendent of the J. N. Adam Hospital at Perrysburg, at a salary of $2,000.

---

Dr. Amos J. Givens of Stamford, Conn., was awarded the degree of LL.D., by Wesleyan University of Middleport, Conn., June 19.

---

Dr. Max Breuer sails for Europe July 18th, to spend some time in Paris, Munich, Heidelberg and Berlin. He will visit the cancer research hospitals in Germany and attend the international congress for Gynecology in Berlin in September .

---

Drs. S. Case Jones, W. W. Percy, C. O. Boswell, E. W. Kennedy, R. D. Richman and Frederick W. Seymour are the Rochester National Guard men attending the Military Medical School at Peekskill, June 22 to 29.

---

Dr. L. Emmett Holt of New York, was in Rochester for several days attending the meetings of the Board of Trustees of the University of Rochester of which he is an alumnus.

---

Dr. Henry H. Covell of Rochester, has just returned from the Chicago Republican Convention.

---

Dr. Henry T. Williams of Rochester, has returned from Newport, R. I.

Dr. W. S. Grimes of Detroit, has moved to 66 Pasadena Ave.

---

Dr. R. Montfort Scheey of Buffalo, read a paper at the American Institute of Homoeopathy at Pittsburg, in June.

---

Unknown persons borrowed Dr. Henry Adsit's (Buffalo) automobile from in front of the 74th Armory, June 18. The automobile was found the next day, uninjured.

---

Dr. Edward J. Meyer of Buffalo, has been elected president of the board of managers of the Public General Hospital, to be erected later.

---

Dr. Earl P. Lothrop has purchased the Lexington Heights Hospital of Buffalo. The present building will be moved to the rear of the lot remodeled into a nurses' home and a modern hospital will be erected.

---

Dr. John F. Fairbairn of Buffalo will spend the summer in Europe.

---

Dr. Loren H. Staples of Buffalo, was in Toronto during the last week of May.

---

Dr. William Gaerner of Buffalo, attended the 10th annual meeting of the United German Societies of New York, in New York City, May 28.

---

Dr. N. Victoria Chappell of Buffalo, sustained a simple, multiple fracture of both bones of the leg, early in May, due to strain in slipping and endeavoring to recover her balance. Her presence of mind in resisting well meant first-aid, undoubtedly prevented the fracture from becoming compound, as she insisted in lying where she fell until temporary splints could be applied. While the case is progressing favorably, Dr. Chappell is still confined to the house.

---

Dr. George S. Dickinson, formerly of Erie, Pa., who has been taking post graduate courses in New York, called on the editor early in June.

---

Dr. A. Crandall Way of Perry, N. Y., formerly of Buffalo, was in Buffalo last week.

